# In situ FRET measurement of cellular tension using conventional confocal laser microscopy in newly established reporter mice expressing actinin tension sensor

**DOI:** 10.1038/s41598-023-50142-z

**Published:** 2023-12-20

**Authors:** Junfeng Wang, Eijiro Maeda, Yuki Tsujimura, Takaya Abe, Hiroshi Kiyonari, Tetsuya Kitaguchi, Hideo Yokota, Takeo Matsumoto

**Affiliations:** 1 Biomechanics Laboratory, Department of Mechanical Systems Engineering, Graduate School of Engineering, Nagoya University, Furo-Cho, Chikusa-Ku, Nagoya, Aichi 464-8603 Japan; 2 RIKEN Center for Advanced Photonics, RIKEN, Wako, Saitama Japan; 3 Laboratory for Animal Resources and Genetic Engineering, RIKEN Center for Biosystems Dynamics Research, Kobe, Hyogo Japan; 4 Laboratory for Chemistry and Life Science, Institute of Innovative Research, Tokyo Institute of Technology, Yokohama, Kanagawa Japan

**Keywords:** Biophysics, Biotechnology, Physics

## Abstract

FRET-based sensors are utilized for real-time measurements of cellular tension. However, transfection of the sensor gene shows low efficacy and is only effective for a short period. Reporter mice expressing such sensors have been developed, but sensor fluorescence has not been measured successfully using conventional confocal microscopy. Therefore, methods for spatiotemporal measurement of cellular tension in vivo or ex vivo are still limited. We established a reporter mouse line expressing FRET-based actinin tension sensors consisting of EGFP as the donor and mCherry as the acceptor and whose FRET ratio change is observable with confocal microscopy. Tension-induced changes in FRET signals were monitored in the aorta and tail tendon fascicles, as well as aortic smooth muscle cells isolated from these mice. The pattern of FRET changes was distinctive, depending on tissue type. Indeed, aortic smooth muscle cells exhibit different sensitivity to macroscopic tensile strain in situ and in an isolated state. This mouse strain will enable novel types of biomechanical investigations of cell functions in important physiological events.

## Introduction

Mechanical forces govern many cellular functions, ranging from embryonic development to etiology of critical diseases. Accordingly, many researchers study how cells sense and respond to extrinsic mechanical loading, and how cells generate mechanical force to regulate the extracellular environment. To monitor the intracellular mechanical environment, several tension sensors using Förster resonance energy transfer (FRET) have been developed, such as vinculin^1^ and actinin tension sensors^2–4^. With these sensors, tension generated in actomyosin units, such as actin stress fibers, can be quantitatively measured, a parameter known as cellular tension. These FRET sensors were introduced into established cell lines and into cells isolated from animal tissues using gene transfection methods. Although basic functionality of these sensors has been validated under culture conditions, our pilot study demonstrated that the success rate of conventional gene transfection was not high (~ 30–40%) with cultured cells and almost zero for cells in isolated tissues. Moreover, expression of mutant genes was not sustained in transfected cells and fluorescence was greatly diminished only one week after transfection, showing its inviability for long-term observations. In addition, there is growing interest in how cells in situ sense mechanical stimulation under load, as in muscles and other load-bearing tissues such as blood vessels and tendons. Such investigations could benefit greatly from reporter mouse lines.

**Figure 1:**
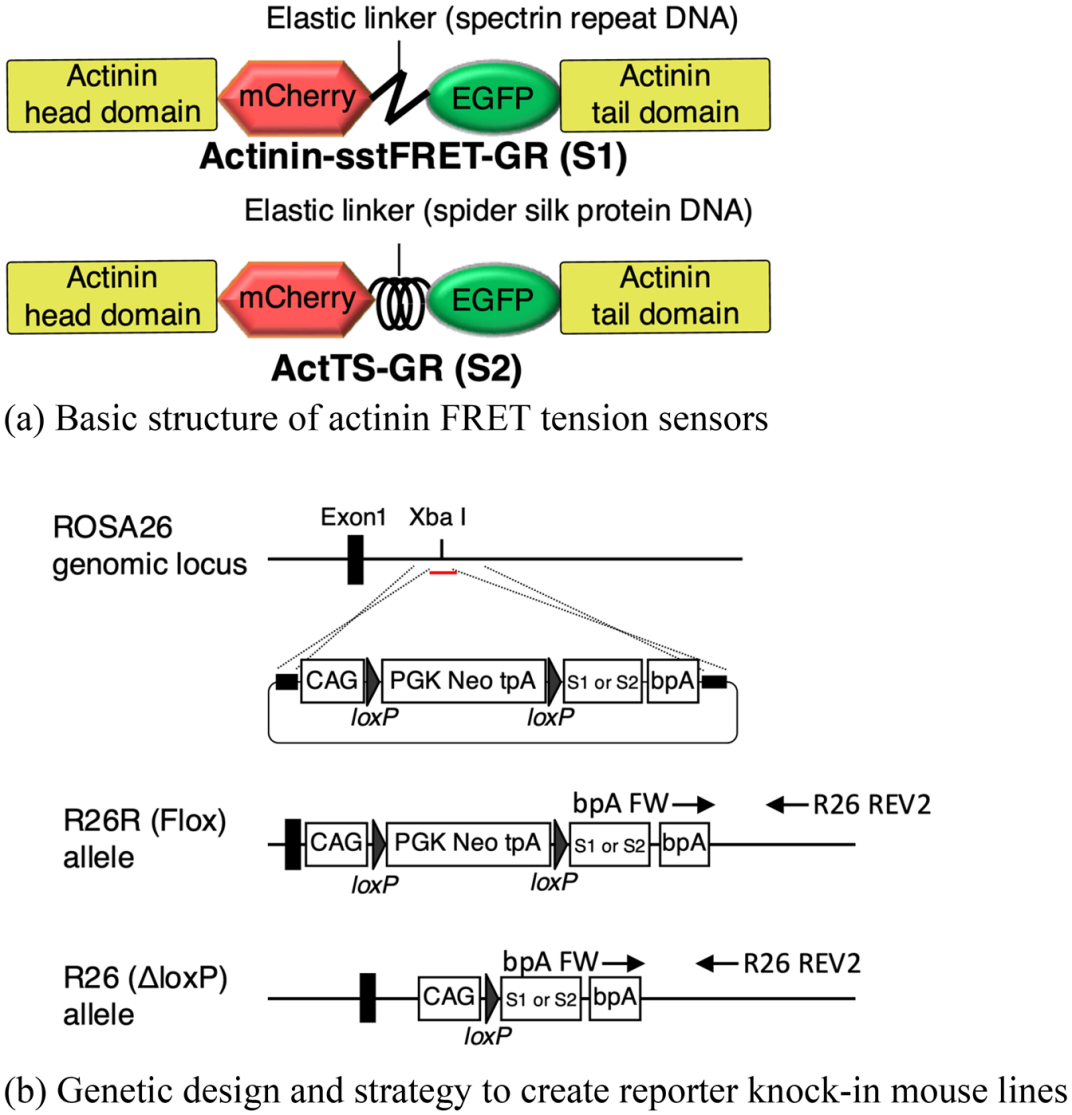
(**a**) Molecular structure of FRET tension sensors Actinin-sstFRET-GR (S1) and ActTS-GR (S2). (**b**) Genetic design and strategy to create reporter knock-in mouse lines expressing FRET tension sensors. Arrows pointing out from “bpA FW” and “R26 REV2” indicate the PCR primer binding sites for genotyping.

Recently, a reporter mouse line was established, expressing a vinculin tension sensor comprising a donor fluorophore, mTFP1, and an acceptor fluorophore, Venus^5^. This study successfully monitored oscillation of intracellular force in mesenchymal stem cells during mandibular arch morphogenesis in mouse embryos, and a FLIM (Fluorescence Lifetime Imaging) system was used to monitor tension signals from those sensors. FLIM is one of the tools capable of precise measurements of FRET efficiency between a pair of fluorophores. A direct comparison of fluorescence of donors and acceptors has also been used to calculate FRET efficiency as a FRET ratio, using a conventional fluorescence/confocal laser scanning microscope^2–4,6–10^. One possible limitation of the vinculin tension sensor^5^ is that in vivo FRET observation has only been carried out with FLIM, and simpler, less-expensive options, such as FRET ratio measurements were not performed. By contrast, we have developed an actinin tension sensor consisting of a donor fluorophore, EGFP, and an acceptor fluorophore, mCherry^3,4^ (Fig. 1a), and we have confirmed that the FRET signal and its tension-related changes can be monitored using a conventional confocal laser scanning microscope. To further enhance the utility of our actinin tension sensor system and to advance mechanobiology research, it would be ideal to have reporter mice expressing our tension sensor systemically.

Therefore, in this study, we established ROSA26 knock-in reporter mouse lines (R26R-S1, R26R-S2, R26-S1 and R26-S2) (Fig. 1b) expressing actinin tension sensors^3,4^ and demonstrated FRET signals in a variety of murine tissues. Using a mechanical loading system mounted on a conventional confocal microscope, tension-induced changes in FRET signals can be monitored in a variety of cells and tissues isolated from these reporter mice and patterns of FRET changes vary according to the tissue type.

## Results

### Robust fluorescence from sensor proteins

**Figure 2:**
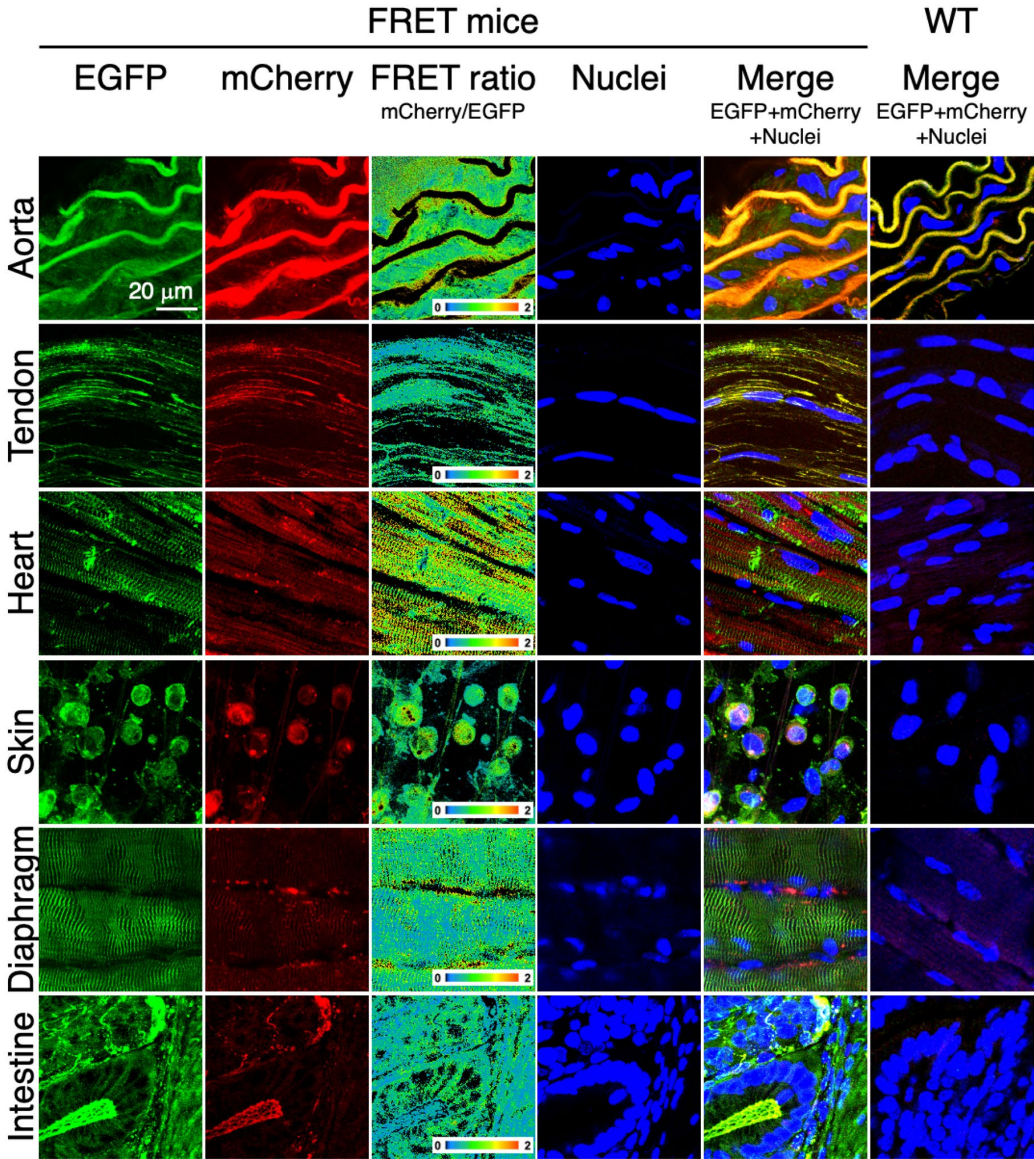
Representative fluorescence images of EGFP and mCherry (excited by FRET from EGFP emission) from S2 tension sensors, color map of FRET ratio distribution, and Hoechst 33,342 staining of cell nuclei in aorta, tendon fascicle, heart, skin, diaphragm, and intestine from R26-S2 mice. Corresponding images from WT mice are also presented.

**Figure 3:**
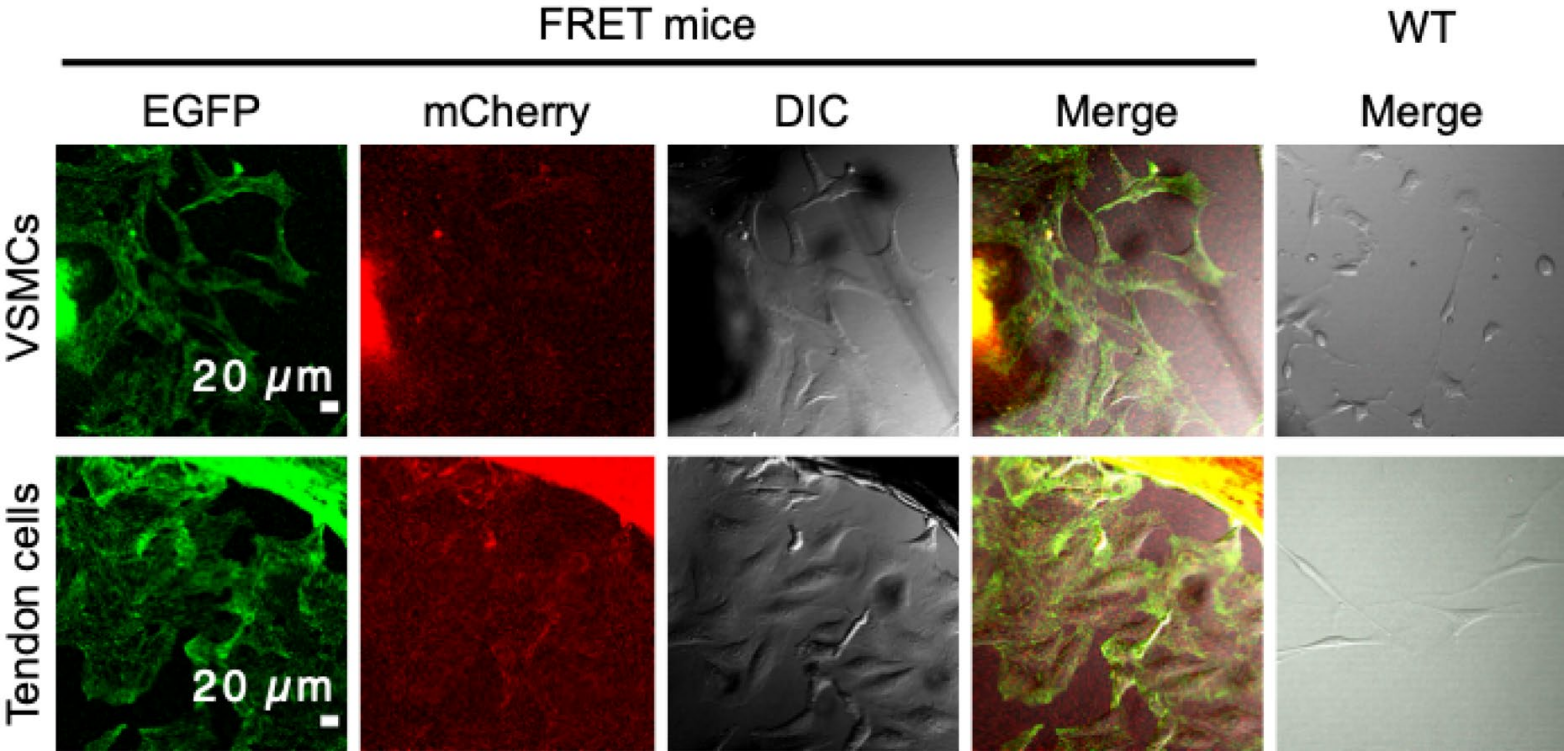
Representative fluorescence images of EGFP and mCherry (excited by FRET from EGFP emission) from S2 tension sensors, and corresponding DIC images from vascular smooth muscle cells and tendon cells from R26-S2 mice. Corresponding images from WT mice are also presented.

First, we examined whether tissues and cells isolated from these reporter mice exhibit fluorescence from the donor and acceptor fluorophores, as well as the FRET phenomenon between them. Aorta, tendon, heart, skin, diaphragm, and intestine, as well as vascular smooth muscle cells (VSMCs) from the aorta and tenocytes from tail tendon were isolated and subjected to confocal microscopy. A robust level of fluorescence was confirmed from EGFP and mCherry expressed in cells from the aforementioned tissues (Fig. 2). Fluorescence from mCherry was emitted via FRET between EGFP and mCherry. In the aorta, VSMCs reside between elastic lamellae (shown as undulating thick lines in the images, visualized by elastin autofluorescence). In R26-S2 mice, there were fluorescent signals between lamellae from EGFP and mCherry in tension sensors; however, no such signals were observed between lamellae in wild-type (WT) mice. In tendon fascicles and other tissues from R26-S2 mice, EGFP signals were demonstrated near and/or between cell nuclei, which were not observed in WT mice. Likewise, cells isolated from the aorta and tendon fascicles also exhibited robust EGFP and mCherry (via FRET) signals (Fig. 3), although no such signals were detected in corresponding WT cells. These results clearly show that tension sensor proteins are expressed and function properly in various tissues from R26-S2 mice, and that the fluorescence level is sufficient to observe using conventional confocal microscopy.

### Acceptor photobleaching experiments

**Figure 4:**
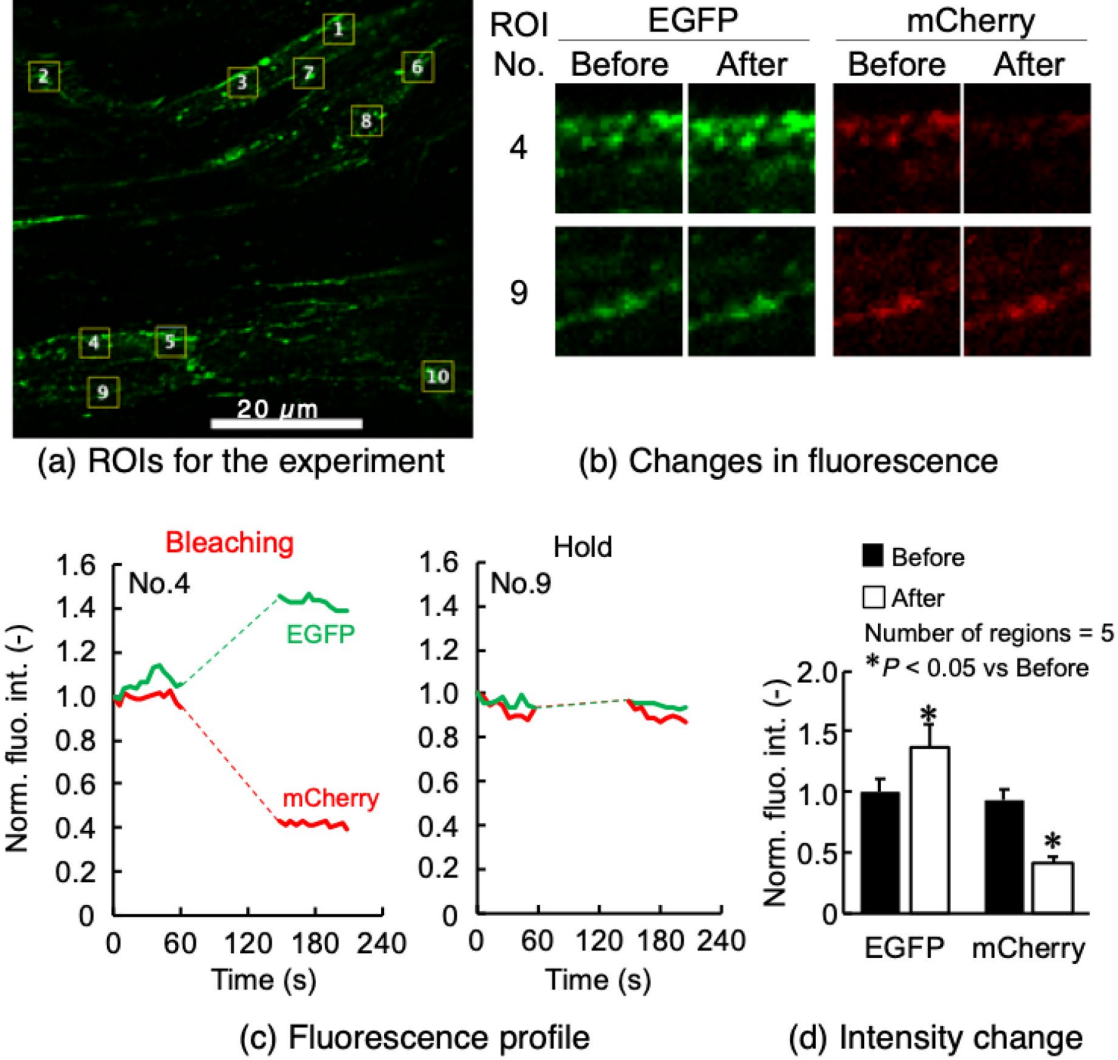
Acceptor photobleaching experiments in tendon fascicle as a representative example. (**a**) Five photobleached ROIs (No. 1–5) and five unbleached ROIs (No. 6–10) are shown as yellow open squares over EGFP fluorescent images. (**b**) Fluorescence changes in EGFP and mCherry in photobleached ROI No.4 and unbleached ROI No.9. (**c**) Fluorescence intensity profile during an experiment in ROIs No.4 (left) and No.9 (right). Intensities were normalized to the values at time 0. (**d**) Comparisons of normalized fluorescence intensity of EGFP and mCherry before and after photobleaching. Data were obtained from S2 tension sensors.

FRET functionality was assessed after photobleaching the acceptor fluorophore, mCherry (Fig. 4). In tendon fascicle, five small ROIs (Regions 1–5) were set in the area where green EGFP fluorescence was observed around cells (Fig. 4a). Another five ROIs (Regions 6–10) were not subjected to photobleaching or imaging during the photobleaching period. In Region1-5, the intensity of mCherry decreased remarkably after photobleaching, while that of EGFP increased relative to the pre-bleaching period (Fig. 4b,c). The increase in EGFP intensity and decrease in mCherry intensity was statistically significant (Fig. 4d). On the other hand, in Regions 6–10 where no photobleaching was performed, the intensity of EGFP and mCherry signals was unchanged. FRET efficiency *E* of the bleaching regions was calculated using the formula *E* = 1 − *I*_pre_/*I*_post_, where *I*_pre_ and *I*_post_ are the intensity of EGFP before and after photobleaching, respectively. The FRET efficiency of tendon was 0.27.

Results from acceptor photobleaching experiments with heart, diaphragm, and aorta are shown in Supplementary Fig. S1. There were statistically significant increases in EGFP intensity and significant decreases in mCherry intensity in photobleaching regions in all specimens examined. The FRET efficiency of heart, diaphragm, and aorta were 0.18, 0.15 and 0.14, respectively.

### Chemical stimulation of isolated FRET cells

**Figure 5:**
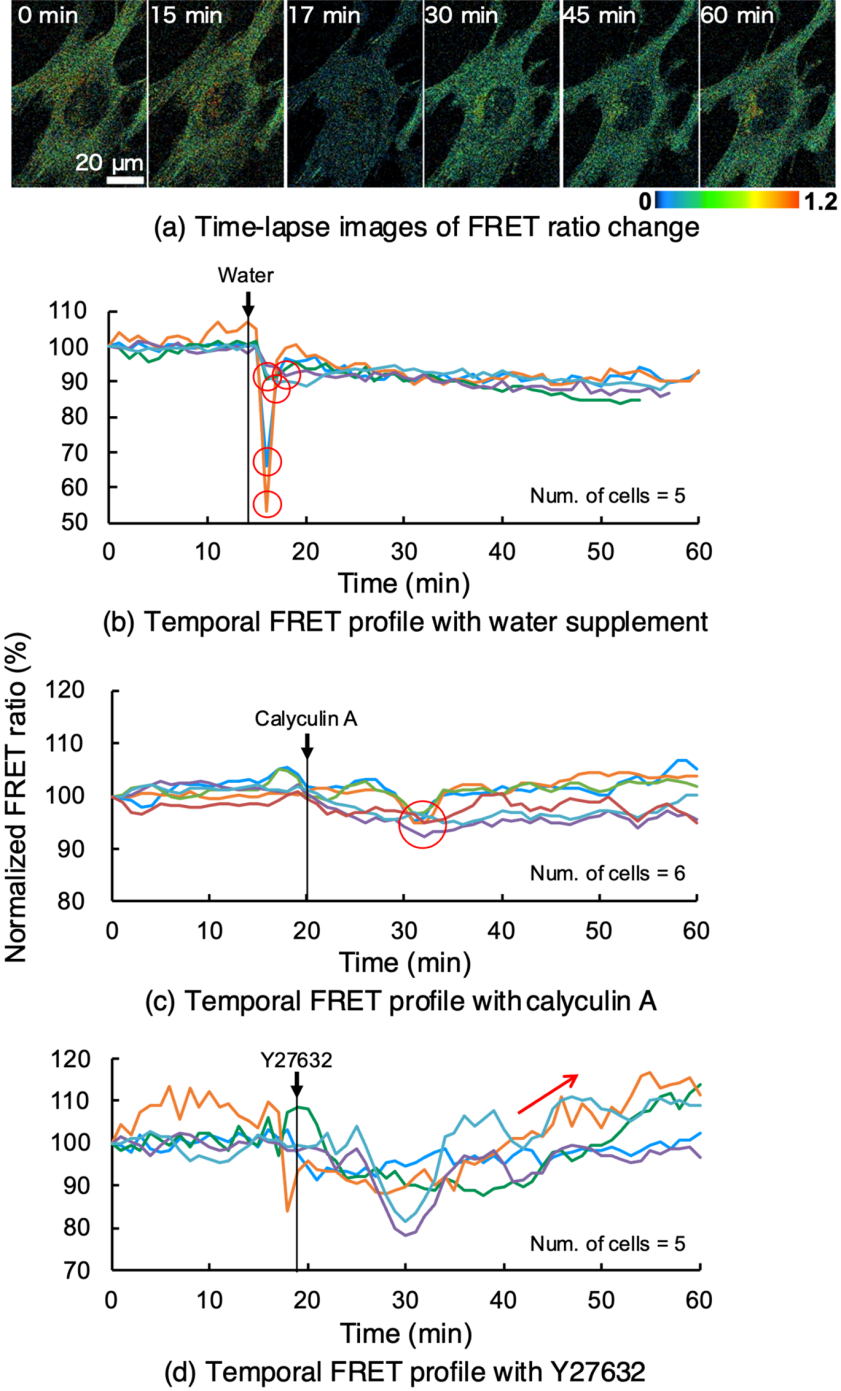
(**a**) Time-lapse images of FRET ratio changes in media diluted with distilled water. Water was introduced to the imaging medium at 14 min. (b-d) Temporal profiles of FRET ratio changes during dilution (**b**), calyculin A (**c**), and Y27632 (**d**). Characteristic effects are highlighted with red open circles as well as a red arrow. Data were obtained from S2 tension sensors.

VSMCs isolated from the aorta in R26-S2 mice exhibited a temporal change in FRET ratio in response to chemical stimulation with distilled water, calyculin A, and Y27632 (Fig. 5). In a passive increase in cellular tension due to reduced osmolality of the culture medium by dilution with distilled water, the FRET ratio dropped sharply by 10–40% immediately, followed by rapid recovery (Fig. 5a,b).

Stimulation with calyculin A induced a ~ 5% decrease in the FRET ratio approximately 10 min after stimulation, followed by recovery to the pre-stimulation level (Fig. 5c).

Y27632 stimulation began to take effect 10 min after administration, increasing the FRET ratio gradually during the remainder of the imaging period. (Fig. 5d).

### Mechanical stimulation of isolated tissues and cells

**Figure 6:**
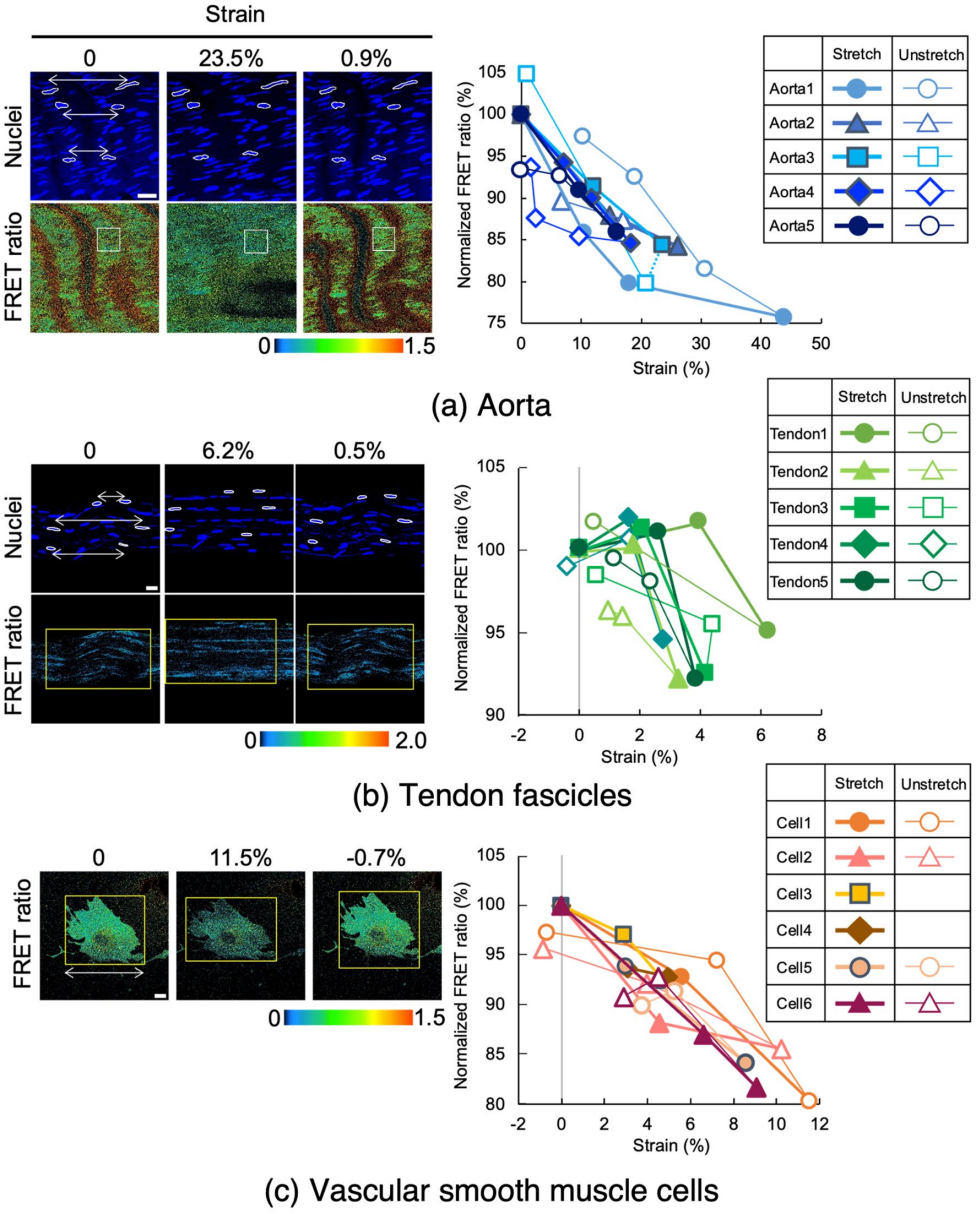
FRET ratio changes in VSMCs in aorta (**a**), tenocytes in tail tendon fascicle (**b**), and isolated VSMCs, in response to macroscopic stretching. Left, snapshots of nuclear fluorescence and the FRET ratio at 0% strain before stretching (left), at the maximum strain (middle), and the final strain during unstretching (right) in one representative experiment. Right, relationships between applied strain and FRET ratio normalized to the value at 0% stretching before stretching. Three specimens were examined in aorta and tendon fascicles and four VSMCs were examined. Unstretching profiles were missed in two of four VSMCs due to technical failures. In (**b**), an initial FRET profile during stretching is shown with a broken line, as this region corresponds to the straightening of crimped collagen fibers and was not included in evaluation of FRET strain sensitivity. Data were obtained from S2 tension sensors.

To examine whether FRET tension sensors can respond to extrinsic mechanical stimulation, tissues and cells isolated from R26-S2 mice were subjected to tensile stretching and unstretching and the FRET ratio was quantified. Each specimen from the aorta was stretched along its circumferential direction from 20 to 50% strain, and the FRET ratio decreased steadily, reaching 15–20% at 25% tensile strain (Fig. 6a). This was followed by an increase in the ratio during unstretching. FRET strain sensitivity (%FRET per %strain) was − 0.70 ± 0.16 (mean ± SD for 5 stretches), − 0.58 ± 0.29 (for 5 unstretches), and − 0.64 ± 0.23 for 10 changes overall.

Tendon fascicles from R26-S2 mice were stretched as much as 5–8% (Fig. 6b). These fascicles exhibited an undulating, crimped pattern in collagen fibers at 0% strain. This was straightened by a small amount of tensile strain (≤ 3%). During this small strain, there was no apparent change in the FRET ratio (Fig. 6b). As fascicles were further stretched, collagen fibers themselves were stretched, and in turn, the ratio decreased 2–5% at 5% strain. The ratio then increased during unstretching of the fascicles. The FRET ratio change during unstretching may involve two steps, as during stretching, although we have not examined this. FRET strain sensitivity was − 5.21 ± 1.70 (for 5 stretches after the first stretching step), − 1.60 ± 0.70 (for 5 unstretches), and − 3.40 ± 2.26 for 10 changes overall.

Cells isolated from R26-S2 mouse tissue also respond to mechanical stimulation. FRET signals in VSMCs from the aorta decreased with an increase in the magnitude of tensile strain and increased with removal of the strain (Fig. 6c). FRET strain sensitivity was − 1.67 ± 0.14 (for 6 stretches), − 1.31 ± 0.31 (for 4 unstretches), and − 1.52 ± 0.31 for 10 changes overall. Comparing the normalized FRET ratio in the aorta at an equivalent level of tensile strain (10%), the decrease in isolated VSMCs was approximately 15%, whereas that in VSMCs in the aorta was 7 to 15% (Fig. 6a), demonstrating a small degree of attenuation of cellular strain in the tissue.

FRET strain sensitivity during stretching differed remarkably among tissues, *i.e.*, aorta and tendon (*P* < 0.0001). In addition, sensitivity of SMC differed significantly between in situ (aorta) and in vitro (VSMCs) (*P* < 0.0001).

## Discussion

We successfully established reporter mice with a pair of fluorophores for FRET-based tension sensing, inserted in actinin amino acid sequences. Functionality of the tension sensor has been confirmed and validated through observation of fluorophore fluorescence, as well as tension-induced changes in FRET in cells in tissues and in culture, under chemical and mechanical stimulation. This mouse line will expand research opportunities in the field of mechanobiology, particularly in regard to how actin-induced cellular tension changes in response to extrinsic mechanical stimulation.

A mouse line with a FRET tension sensor has already been reported by Tao et al.^5^. Fluorophore selection is one of the advantages of our strain over that of Tao et al.^5^. Our FRET fluorophores, EGFP and mCherry, can be observed with conventional confocal laser microscopy^3,9^, whereas the other FRET system employing mTFP1 and Venus requires FLIM^1,5^. Although FLIM provides precise evaluation of FRET efficiency without the need to consider fluorescence bleed-through^11^, the microscope system is very expensive; thus, utilization of the system may be limited. In the present study, despite using a conventional confocal microscope, changes in FRET ratios in response to alterations in cellular tension were clearly observed. This indicates that our FRET mice are a more economical option for those without access to expensive microscopy facilities.

Since the design of this murine FRET tension sensor is the same as that used in our previous studies^3,9^, the results obtained here agreed well with those obtained previously. In the present experiment with low osmolarity to increase VSMC cellular tension, the FRET ratio started dropping immediately after dilution of the media with water, achieving a 10–40% decrease in 2 min (Fig. 5b). Likewise, in our previous experiments, the FRET ratio of FRET sensor-transfected MC3T3-E1 cells similarly started dropping immediately after addition of water, decreasing 20% in 4 min^3^. Although there were small differences in speed and magnitude of maximal FRET induction, which may be due to differences in tissue location or relative dilution, as well as the FRET tension sensor expression level in single cells, responsiveness and overall FRET changes were consistent between the two studies.

The wall of rat thoracic aorta deforms greatly in the circumferential direction under intraluminal pressurization from 0 to 120 mmHg^12,13^ as well as in mice^14^. When calculating strain level within a physiological pressure range (70–120 mmHg), aortic diameter increased approximately 5–10%^13^. An experimental study reported that the level of longitudinal strain in aortic SMCs is smaller than the circumferential strain^15^. Our present results demonstrated that VSMCs in aortic tissue exhibited a decrease in FRET ratio of ~ 10–15% when subjected to macroscopic 10% tensile strain in the circumferential direction (Fig. 6a). Isolated VSMCs exhibited a 15% decrease in the FRET ratio when stretched 10% (Fig. 6c), suggesting that FRET strain sensitivity of VSMC differs between in situ and isolated states. Sugita et al. compared the level of circumferential strain between smooth muscle layers and actin stress fibers in VSMCs in isolated descending thoracic aorta from mice under intraluminal pressure at 120 mmHg and showed that the strain in actin stress fibers in VSMCs in the direction of the fiber axis was half the strain in the smooth muscle layers in the circumferential direction of the aorta^15^. This strain attenuation was mainly because stress fibers in VSMCs in the aorta were inclined 15° from the circumferential direction of the aorta, preventing cells from being exposed to a large amount of strain. Therefore, in the present study, the level of tensile strain to which VSMCs in the aorta were exposed was supposed to be less than the tensile strain macroscopically applied to the aorta, resulting in less cellular and cytoskeletal deformation, and in turn, reduced FRET strain sensitivity. In the case of isolated VSMCs, cells were directly exposed to macroscopic mechanical strain, resulting in greater cellular and cytoskeletal deformation and larger FRET strain sensitivity compared to VSMCs in situ exposed to the same amount of macroscopic strain.

Tendon deformation during body movements at a physiological level (not heavy activity), equivalent to 3–4% tensile strain, occurs mainly by straightening of crimped collagen fibers, corresponding to the “toe region” in stress–strain relationships^16^. With application of a small macroscopic strain to tendon, i.e., 1–2%, microscopic local strain in collagen matrix and nuclear strain are much smaller than the applied macroscopic strain^17^. Current findings from tendon fascicle experiments suggest that the tenocyte actin cytoskeleton may be not deformed, or that cellular tension does not change, during the initial stage of tendon deformation. A relatively larger amount of strain, at least 4%, is required to induce an increase in cellular tension and possibly to activate the actin cytoskeletal mechanotransduction pathway for upregulation of tenocyte anabolic functions, agreeing with previous ex vivo experiments examining mechanoregulation of tenocyte functions^18,19^.

The difference in FRET ratio-strain profiles between tenocytes in situ and VSMCs in situ may reflect the extracellular matrix environment in which these cells reside. In tendon, tenocytes that reside among collagen fibers aligned in parallel may attach firmly to collagen fibers, so that tenocytes were not stretched and the FRET ratio did not decrease until crimped collagen fibers were straightened and began to be stretched. On the other hand, VSMCs which reside between elastic lamellae are under tension even when no mechanical loading was applied to the aorta and elastic lamellae surrounding VSMCs were undulated^20^, enabling these cells to respond immediately once a mechanical load was applied. This may explain why the FRET ratio in VSMCs in situ decreased even under small strains. Although the exact mechanisms underlying strain responsiveness between tendon and artery are not clear, we speculate that VSMCs and elastic lamellae share load-bearing responsibility and the same level of tension is generated in both components under physiological blood pressure, whereas collagen fibers assume the main load-bearing function of tendons during movement, and that tenocyte contractility contributes little. Nonetheless, tenocyte cellular tension itself also serves as a “mechanostat” that can sense extrinsic mechanical stimulation via changes in the tension level^21^.

Differences in FRET strain sensitivity between tendon and aorta is attributed to the amount of physiological deformation in the two tissues. That is, the aorta deforms 10% circumferentially under physiological pressure, whereas tendons generally deform 4% in the longitudinal direction during physiological joint motion, in which collagen fibers stretch only 1–2%. This difference corresponds well to a gross change in the FRET ratio in the physiological range in these tissues: − 0.59 × 10 = − 6% FRET change in the aorta versus − 4.17 × 1.5 = − 6.2% FRET change in tendon. This coincidence may indicate that the amount of deformation of actin fibers in physiological mechanical behavior is preserved in load-bearing soft tissues. In addition, tendon exhibits a fast response to mechanical alteration, decreasing tensile strength and elastic modulus to 50% of their normal levels after stress shielding for 1 week^22^. This was associated with a 30% increase in cross-sectional area. Similarly, a 50% decrease in elastic modulus and a 75–100% increase in the cross-sectional area have been observed in isolated tendon fascicles cultured for 1 week^23,24^. However, this was not the case in the aorta, in which the initial elastic modulus of rabbit common carotid artery did not deteriorate after 1 week of culture without mechanical loading^25^. Such a drastic response in tendon could be relevant to its high strain sensitivity.

Although our main objective was to develop a transgenic mouse strain expressing FRET-based actinin-tension-sensor proteins and to confirm their tension-sensing functions, one of the limitations of the present study is that the FRET ratio was calculated solely on the ratio of the fluorescence intensity of the acceptor (mCherry) divided by that of the donor (EGFP). As mentioned above, a precise FRET ratio has to be measured using an FLIM system or a method considering possible fluorescence bleed-through^11,26^. Nonetheless, the current method of FRET ratio calculation was sufficient to achieve our objective, as changes in the ratio were clearly confirmed both by chemical and mechanical manipulations of cellular tension. Indeed, we have estimated the maximum effect of possible fluorescence bleed-through on values of normalized FRET ratio change, and it was less than 10% in our tensile stretching experiments (Supplementary Material S1). Therefore, we believe that the current method of FRET ratio calculation is reliable.

All FRET measurements in the present study were performed at room temperature, which is the second limitation. At room temperature, myosin may not be activated as much as at 37°C; thus, FRET recovery during unloading could be insufficient. Another limitation is that we observed FRET in actinin as a tension indicator. Because actinin integrates actin filaments into stress fibers as actin-binding protein, its FRET signal does not reflect the force applied to and/or generated in stress fibers. Therefore, although we are able to detect changes in the tension within stress fibers, determination of the absolute value of tension is still challenging, requiring further experiments such as calibrations under a variety of conditions.

In conclusion, we have successfully developed a transgenic mouse strain expressing FRET-based actinin-tension-sensor units incorporating EGFP and mCherry, and confirmed their responsiveness to changes in cellular tension using conventional confocal laser scanning microscopy. Moreover, there were distinctive FRET strain sensitivities in different tissues. This suggests that deformation of actin fibers in cells at the level of macroscopic tissue strain and the extent of subsequent mechanical responses, depends on tissue types. This mouse strain will enable researchers in mechanobiology and biomechanics to investigate mechanical aspects of cell functions in important physiological events.

## Online methods

### Structure of tension sensors

In this study, we used two types of FRET-based tension sensors, Actinin-sstFRET-GR^3^ (denoted as S1 hereafter, Addgene plasmid #83416; http://n2t.net/addgene:83416; RRID: Addgene_83416) and ActTS-GR^4^ (S2, Addgene plasmid #79774; http://n2t.net/addgene:79774; RRID:Addgene_79774), to produce two series of ROSA26 reporter knock-in mice (Fig. 1a,b). These two sensors have similar structure except for their linker proteins. S1 uses a DNA fragment of a spectrin repeat as the linker protein whereas S2 uses a DNA fragment of spider flagelliform silk protein. The DNA fragment of each linker was cloned into the EcoRI/BamHI site of a plasmid. DNA fragments of mCherry (AgeI/EcoRI) and EGFP (BamHI/NotI) were inserted into the N and C-termini of the linker to create a sensor module. This module was fused between the actinin-head (1–300 aa) and actinin-tail domains (301–892 aa). EGFP and mCherry were selected as the FRET fluorophore pair because FRET signals from both fluorophores can be observed with excitation at 488 nm, which is available in conventional fluorescence microscopes, as well as confocal microscopes.

In this paper, we described our experimental results from reporter mice expressing S2 sensors, unless otherwise noted. This is because reporter mice expressing S2 sensors were raised successfully first, and therefore, the data presented in this paper were obtained with them. In selected experiments, we also used the mice expressing S1 sensors and confirmed that the two sensors exhibited similar phenomena. Therefore, there should be few differences in their cellular tension-sensing capabilities.

### Generation of ROSA26 reporter knock-in mice

The conditional R26R-S1 (Accession No. CDB0054E: https://large.riken.jp/distribution/reporter-mouse.html) and R26R-S2 (Accession No. CDB0055E) knock-in mice were generated by CRISPR/Cas9-mediated genome editing in C57BL/6N zygotes as previously described^27^. For CRISPR-mediated knock-in, donor vectors consisting of homology arms, CAG promoter^28^, a loxP-flanked STOP sequence (PGK Neo tpA)^29^, sensor sequence (S1 or S2), and poly-A sequence (bpA) were generated to insert the cassette into the ROSA26 locus. After microinjection, F0 mice were screened by PCR, and the resulting knock-in mice were crossed with wild-type (C57BL/6N) to obtain the next generation and were crossed with the same littermate to generate the homozygous mice. PCR primers were used as follows; R26 FW1 (5′-GCT CCT CAG AGA GCC TCG GCT AGG-3′) and CAG REV (5′-CAA TGT CGA CCT CGA GGG-3′) for 5′-side of R26R allele (1.2 kbp), bpA FW (5′-GGG GGA GGA TTG GGA AGA CAA TAG C-3′) and R26 REV1 (5′-AGA ACT GCA GTG TTG AGG-3′) for 3′-side of R26R allele (0.76 kbp for S1 and 0.71 kbp for S2). To establish the conventional R26-S1 (Accession No. CDB0362E: https://large.riken.jp/distribution/reporter-mouse.html) and R26-S2 (Accession No. CDB0363E) that ubiquitously express the FRET sensor, R26R mice were crossed with a CAG-Cre transgenic mouse (RBRC01828, RIKEN BioResource Research Center)^30^, and homozygous R26 mice that is Cre transgene positive were obtained, respectively. The routine genotyping PCR was performed using the following primers: P1 (5′-GTT TCA CTG GTT ATG CGG CGG-3′) and P2 (5′-TTC CAG GGC GCG AGT TGA TAG-3′) for the detection of the Cre transgene (450 bp)^30^, R26 FW2 (5′- TCC CTC GTG ATC TGC AAC TCC AGT C-3′) and R26 REV2 (5′- AAC CCC AGA TGA CTA CCT ATC CTC C-3′) for the wild type allele (217 bp), and bpA FW and R26 REV2 for R26R and R26 alleles (342 bp for S1 and 297 bp for S2).

All procedures performed on these animals were approved by the Animal Care Committee of Nagoya University Graduate School of Engineering (Nos. 19–2, 20–5, GS220011), the Nagoya University Committee for Recombinant DNA Experimentation (No.17–3), the Institutional Animal Care and Use Committee of RIKEN Kobe Branch (A2001-03), and a genetic recombinant experiment safety committee of RIKEN Kobe Branch (H17-04). All methods were performed in accordance with the ARRIVE guidelines^31^ as well as the relevant guidelines and regulations of the above committees including the Guide for Animal Experimentation, Nagoya University.

To perform experiments described below, tissue and cell specimens were isolated from male R26 reporter mice at 4 weeks or older. Mice were sacrificed with CO_2_ gas, and the thoracic aorta, heart, tail tendon, skin (dermis), diaphragm, and intestines were removed and kept in phosphate buffered saline (PBS) at 4°C until use. For cell isolation, a portion of the aorta or tail tendon fascicles was cut into small pieces. These pieces were cultured in Dulbecco's modified eagle’s medium (DMEM, Wako, Japan) supplemented with 10% fetal bovine serum (Biowest, France), penicillin (100 unit/mL), and streptomycin (100 μg/mL, Sigma, USA) in a plastic dish to allow smooth muscle cells in aortic media or tenocytes to migrate from the tissue fragments to the bottoms of the dishes. Isolated cells were maintained until passage 2 before storage at − 80°C or use in experiments. Wild-type tissues and cells were isolated from C57BL/6N mice (male, 18-week-old) separately purchased using essentially similar procedures described above.

### Confirmation of FRET in tissues and cells isolated from R26 reporter mice

To confirm that EGFP and mCherry are functionally expressed, isolated tissues and cells were observed with a confocal laser scanning microscope. Tissue specimens were prepared from aorta, tail tendon, heart, skin, diaphragm, and intestine in hydrated conditions. Aortic specimens were prepared by embedding isolated aortic segments with a length of 5 mm in an agar gel and cutting out rings 200 µm in length. Tendon specimens were prepared by teasing out tendon fascicles. Heart specimens were prepared by embedding the left ventricle in an agar gel and cutting 200-µm transverse sections. Skin specimens were prepared by dissecting abdominal skin, removing subcutaneous tissues. These were observed from the dermal side. Intestine specimens were prepared by embedding the whole intestine in an agar gel and cutting it into 200-µm rings. All cutting procedures were performed with a microslicer (DTK-1000, Dosaka-EM, Japan).

For tissue observation, cell nuclei were labeled with Hoechst 33,342 (Molecular Probes, USA). Tissue samples were placed on glass-bottomed dishes and covered with a coverslip while kept hydrated with PBS at room temperature (25°C). They were observed with a confocal laser scanning microscope (FV1200 + IX81, Olympus, Japan) and a 40 × silicone immersion objective (UPLSAPO40XS, N.A. = 1.25, Olympus). Cell nuclei were observed at 2% power with an excitation laser at 405 nm and emission was detected between 430 and 470 nm. EGFP fluorophores were excited at 2% power of an excitation laser at 488 nm and emission was detected between 505 and 525 nm. Emission from mCherry was detected between 560 and 660 nm. For cell observation, tissue samples were laid on plastic dishes and incubated in culture medium to isolate cells. Cells attached to the plastic dish were observed in similar fashion with a 20 × objective lens (NA = 0.45). A series of z-stack images of tissues and cells were obtained at a z-interval of 1 µm for tissues and 5 µm for cells, respectively. Maximum intensity projection images were also created following acquisition with ImageJ/Fiji (ver.1.53c, NIH, USA). The same observations were also carried out with wild-type tissues and cells (C57BL/6N mice) as negative controls.

Acceptor photobleaching experiments were also performed to confirm whether FRET occurs between two fluorophores in our tension sensor, EGFP and mCherry. Theoretically, inactivation of an acceptor by photobleaching increases the fluorescence of the donor if FRET occurs. In contrast, in the absence of FRET, photobleaching of the acceptor has no effect on donor fluorescence intensity. Acceptor photobleaching was performed on specimens prepared from tail tendon fascicles, the aorta, heart, and diaphragm from transgenic mice as described above, using a confocal laser microscope (LSM880, Carl Zeiss, Germany) and a 63 × oil immersion objective (PLAN-APOCHROMAT, N.A. = 1.40, Carl Zeiss). A 543-nm excitation laser at 100% power was applied to small rectangular regions of interest (ROIs) 100 times, ranging from 1 µm × 3 µm to 4 µm × 4 µm square regions, set within cell body on the tissues to photobleach mCherry. The fluorescence intensity of EGFP (495–550 nm) and mCherry (580–624 nm) was measured before and after photobleaching. To obtain negative control data, the same procedure was performed in regions without cells, or no photobleaching was performed in regions around cells, but the sample was held for a period corresponding to the bleaching period.

### Tension sensor FRET functionality test

#### Chemical modification of cellular tension

To test whether tension sensors respond to changes in intracellular and extracellular mechanical environments, chemical and mechanical stimulation were applied to vascular smooth muscle cells isolated from aortas of R26 reporter mice. To examine chemically induced changes in the intracellular mechanical environment, distilled water, calyculin A, or ROCK inhibitor Y27632 was applied to cells. Distilled water and calyculin A were applied to increase cellular tension passively and actively, respectively, whereasY27632 was applied to decrease cellular tension actively. Cells were seeded on a glass-bottomed dish coated with fibronectin (100 μg/mL, Sigma) and cultured for 24 h before stimulation. Time lapse observation of EGFP and mCherry fluorescence was performed with a confocal microscope (Zeiss) and 63 × oil immersion lens at 1-min intervals for 1 h at room temperature. A volume of distilled water (1 mL) at room temperature equivalent to half the culture medium was added to dilute the medium and to increase cellular tension under exposure to hypotonic stress. For active elevation of cellular tension, calyculin A (20 µM) was supplied to the medium at a final concentration of 10 µM. To actively decrease cellular tension, Y27632 (40 nM) was added to the medium at a final concentration of 20 nM. In each experiment, the stimulating reagent was added at a designated time point during the 1-h imaging period.

#### Application of tensile strain to tissues and cells

To examine responses of tension sensor proteins to mechanically induced cellular tension, the aorta, tendon fascicles and vascular smooth muscle cells isolated from the aorta were subjected to tensile stretching and unstretching while FRET observation was performed. A rectangular aortic specimen was prepared by cutting the isolated aorta into 1-mm rings, which were then cut transversely, resulting in a 1 mm × 2.5 mm (circumferential length) specimen. After staining of cell nuclei in Hoechst 33,342 in PBS, specimens were attached to a tensile tester (STB 150W NK, Strex, Japan) using custom-made jigs^32^ (Supplementary Fig. S2), parallel to the stretching direction and the intima side facing the objective lens. The testing device was mounted on the motorized stage of the confocal laser microscope system (Zeiss) using the 63 × objective lens. Tensile strain was applied up to ~ 40% at room temperature while the specimen was kept hydrated with PBS. Positional changes of three pairs of cell nuclei in fluorescent images in each strain step were manually recorded and used for local strain calculations relative to their initial, 0% strain positions. At each strain step, a z-series of fluorescent images of cell nuclei, EGFP, and mCherry was obtained with a z-interval of 0.5 µm. Cell nuclei were imaged with a 405-nm wavelength laser. Fluorescence emissions of EGFP (emission 495–550 nm) and mCherry (emission 580–624 nm) were obtained using each designated detector on the same timing only with a 488-nm wavelength laser at 2% power. After reaching the prescribed maximum level of tensile strain, the tissue was unloaded to 0% strain, and fluorescent images were again obtained.

Tendon fascicles were teased from mouse tails. Experimental samples 20 mm in length were cut from fascicles and labelled with Hoechst 33342 in PBS. Specimens were attached to the same tensile tester using a set of custom-made jigs. Tensile strain ≤ 10%was applied and fluorescent images of cell nuclei, EGFP, and mCherry were obtained as described above at room temperature. Cell nuclei were used as strain markers. Taking collagen fiber dynamics into account, tendon fascicles were first stretched at ~ 2 to 4% tensile strain to straighten crimped collagen fibers and fluorescent images were acquired. This was followed by additional stretching of tendon fascicles and acquisition of fluorescent images.

VSMCs were first seeded in a PDMS chamber (STB-CH-0.2, Strex) in an incubator and allowed to attach to the elastic membrane for 24 h. Before the stretching experiment, cells were labelled with Hoechst 33342. The chamber was attached to the same tensile tester and mounted onto the confocal microscope (Zeiss). Tensile strain was applied to 20%, and fluorescent images of cell nuclei, EGFP, and mCherry were obtained as described above at room temperature. The length of the cell body was also measured to calculate the global strain on the cell.

Changes in the FRET ratio by application of mechanical strain were determined from images obtained at each strain step. In each set of z-stack images, the z-position in which the fluorescent image of EGFP can be seen best was selected. In the selected z-position, the FRET ratio in the region where cell nuclei were visible was calculated by dividing the signal intensity of the acceptor *I*^mCherry^ by that of the donor *I*^EGFP^ (*I*^mCherry^/*I*^EGFP^). Relationships between the FRET ratio and local tissue strain were examined, where the latter was calculated from cell nuclear positions. We expected that the FRET ratio would decrease during tensile stretching and would increase during unstretching.

### Statistical analysis

Comparisons between two groups were performed with Student’s t-tests (two-sided) using the statistical language R (version 4.3.0). The assumption of the normal distribution of data and the equality of variance were confirmed with Shapiro–Wilk tests and Bartlett tests, respectively. In all analyses, the statistical significance level was set at *P* < 0.05.

## Supplementary Information

Supplementary Information.

## Data Availability

The datasets generated and analyzed during the current study are available from the corresponding author on reasonable request.

## References

[1] Grashoff, C. *et al.* Measuring mechanical tension across vinculin reveals regulation of focal adhesion dynamics. *Nature* **466**, 263–266 (2010). 20613844 10.1038/nature09198 PMC2901888

[2] Meng, F. & Sachs, F. Visualizing dynamic cytoplasmic forces with a compliance-matched FRET sensor. *J. Cell Sci.* **124**, 261–269 (2011). 21172803 10.1242/jcs.071928 PMC3010192

[3] Wang, J. *et al.* Observations of intracellular tension dynamics of MC3T3-E1 cells during substrate adhesion using a FRET-based actinin tension sensor. *J. Biomech. Sci. Eng.* **11**, 16–00504 (2016).

[4] Yamashita, S., Tsuboi, T., Ishinabe, N., Kitaguchi, T. & Michiue, T. Wide and high resolution tension measurement using FRET in embryo. *Sci. Rep.* **6**, 28535 (2016). 27335157 10.1038/srep28535 PMC4917836

[5] Tao, H. *et al.* Oscillatory cortical forces promote three dimensional cell intercalations that shape the murine mandibular arch. *Nat. Commun.* **10**, 1703 (2019). 30979871 10.1038/s41467-019-09540-z PMC6461694

[6] Knorr, J. M., Jackson, J., Batie, M. R., Narmoneva, D. A. & Jones, D. C. Application of strain and calibration of Förster Resonance Energy Transfer (FRET) emission for in vitro live cell response to cytoskeletal deformation. *J. Biomech.* **49**, 3334–3339 (2016). 27589930 10.1016/j.jbiomech.2016.08.023

[7] Kim, T. J. *et al.* Dynamic visualization of α-catenin reveals rapid, reversible conformation switching between tension states. *Curr. Biol.* **25**, 218–224 (2015). 25544608 10.1016/j.cub.2014.11.017 PMC4302114

[8] Taylor-Weiner, H., Ravi, N. & Engler, A. J. Traction forces mediated by integrin signaling are necessary for definitive endoderm specification. *J. Cell Sci.* **128**, 1961–1968 (2015). 25908864 10.1242/jcs.166157 PMC4457159

[9] Wang, J. *et al.* A novel FRET analysis method for tension dynamics in a single actin stress fiber: Application to MC3T3-E1 cells during movement on a substrate. *J. Biorheol.* **33**, 21–26 (2019).

[10] Meng, F., Suchyna, T. M. & Sachs, F. A fluorescence energy transfer-based mechanical stress sensor for specific proteins in situ. *FEBS J.* **275**, 3072–3087 (2008). 18479457 10.1111/j.1742-4658.2008.06461.x PMC2396198

[11] Abraham, B. G. *et al.* Fluorescent protein based FRET pairs with improved dynamic range for fluorescence lifetime measurements. *PLoS One* **10**, e0134436 (2015). 26237400 10.1371/journal.pone.0134436 PMC4523203

[12] Matsumoto, T., Tsuchida, M. & Sato, M. Change in intramural strain distribution in rat aorta due to smooth muscle contraction and relaxation. *Am. J. Physiol. Circ. Physiol.* **271**, H1711–H1716 (1996). 10.1152/ajpheart.1996.271.4.H1711 8897968

[13] Maeda, E., Ando, Y., Takeshita, K. & Matsumoto, T. Through the cleared aorta: three-dimensional characterization of mechanical behaviors of rat thoracic aorta under intraluminal pressurization using optical clearing method. *Sci. Rep.* **12**, 8632 (2022). 35606390 10.1038/s41598-022-12429-5 PMC9126909

[14] Sugita, S., Kato, M., Wataru, F. & Nakamura, M. Three-dimensional analysis of the thoracic aorta microscopic deformation during intraluminal pressurization. *Biomech. Model. Mechanobiol.* **19**, 147–157 (2020). 31297645 10.1007/s10237-019-01201-w PMC7005079

[15] Sugita, S., Mizuno, N., Ujihara, Y. & Nakamura, M. Stress fibers of the aortic smooth muscle cells in tissues do not align with the principal strain direction during intraluminal pressurization. *Biomech. Model. Mechanobiol.* **20**, 1003–1011 (2021). 33515313 10.1007/s10237-021-01427-7 PMC8154808

[16] Wang, J. H. C. Mechanobiology of tendon. *J. Biomech.* **39**, 1563–1582 (2006). 16000201 10.1016/j.jbiomech.2005.05.011

[17] Screen, H. R. C., Lee, D. A., Bader, D. L. & Shelton, J. C. Development of a technique to determine strains in tendons using the cell nuclei. *Biorheology* **40**, 361–368 (2003). 12454427

[18] Maeda, E., Shelton, J. C., Bader, D. L. & Lee, D. A. Differential regulation of gene expression in isolated tendon fascicles exposed to cyclic tensile strain in vitro. *J. Appl. Physiol.* **106**, 506–512 (2009). 19036888 10.1152/japplphysiol.90981.2008

[19] Maeda, E., Shelton, J. C., Bader, D. L. & Lee, D. A. Time dependence of cyclic tensile strain on collagen production in tendon fascicles. *Biochem. Biophys. Res. Commun.* **362**, 399–404 (2007). 17719009 10.1016/j.bbrc.2007.08.029

[20] Matsumoto, T., Goto, T., Furukawa, T. & Sato, M. Residual stress and strain in the lamellar unit of the porcine aorta: experiment and analysis. *J. Biomech.* **37**, 807–815 (2004). 15111068 10.1016/j.jbiomech.2003.08.014

[21] Arnoczky, S. P. *et al.* Loss of homeostatic strain alters mechanostat ‘set point’ of tendon cells in vitro. *Clin. Orthop. Relat. Res.* **466**, 1583–1591 (2008). 18459031 10.1007/s11999-008-0264-x PMC2505257

[22] Yamamoto, N. *et al.* Effects of stress shielding on the mechanical properties of rabbit patellar tendon. *J. Biomech. Eng.* **115**, 23–28 (1993). 8445894 10.1115/1.2895466

[23] Yamamoto, E., Tokura, S. & Hayashi, K. Effects of cyclic stress on the mechanical properties of cultured collagen fascicles from the rabbit patellar tendon. *J. Biomech. Eng.* **125**, 893–901 (2003). 14986416 10.1115/1.1634286

[24] Yamamoto, E., Iwanaga, W., Miyazaki, H. & Hayashi, K. Effects of static stress on the mechanical properties of cultured collagen fascicles from the rabbit patellar tendon. *J. Biomech. Eng.* **124**, 85–93 (2002). 11871609 10.1115/1.1427924

[25] Matsumoto, T., Okumura, E., Miura, Y. & Sato, M. Effect of smooth muscle cells on the mechanical response of rabbit carotid arteries in culture. *JSME Int. J Ser. C Mech. Syst. Mach. Elem. Manuf.* **42**, 513–520 (1999).

[26] Periasamy, A., Wallrabe, H., Chen, Y. & Barroso, M. *Chapter 22 Quantitation of Protein-Protein Interactions. Confocal FRET Microscopy*. *Methods in Cell Biology* **89**, 569–598 (Elsevier Inc., 2008). 10.1016/S0091-679X(08)00622-5 19118691

[27] Abe, T., Inoue, K., Furuta, Y. & Kiyonari, H. Pronuclear microinjection during S-phase increases the efficiency of CRISPR-Cas9-assisted knockin of large DNA donors in mouse zygotes. *Cell Rep.* **31**, 107653 (2020). 32433962 10.1016/j.celrep.2020.107653

[28] Niwa, H., Yamamura, K. & Miyazaki, J. Efficient selection for high-expression transfectants with a novel eukaryotic vector. *Gene* **108**, 193–199 (1991). 1660837 10.1016/0378-1119(91)90434-d

[29] Srinivas, S. *et al.* Cre reporter strains produced by targeted insertion of EYFP and ECFP into the ROSA26 locus. *BMC Dev. Biol.* **1**, 1–8 (2001). 11299042 10.1186/1471-213X-1-4 PMC31338

[30] Matsumura, H., Hasuwa, H., Inoue, N., Ikawa, M. & Okabe, M. Lineage-specific cell disruption in living mice by Cre-mediated expression of diphtheria toxin A chain. *Biochem. Biophys. Res. Commun.* **321**, 275–279 (2004). 15358172 10.1016/j.bbrc.2004.06.139

[31] Percie du Sert, N. *et al.* The ARRIVE guidelines 20: Updated guidelines for reporting animal research. *PLoS Biol.* **18**, e3000410 (2020). 32663219 10.1371/journal.pbio.3000410 PMC7360023

[32] Fan, Y., Wang, J., Kim, J., Maeda, E. & Matsumoto, T. Dependency of deformation of cell nucleus on stretch direction of tissue: Relation to anisotropic response of aortic media to hypertension. *J. Mech. Behav. Biomed. Mater.* **133**, 105326 (2022). 35779487 10.1016/j.jmbbm.2022.105326

