# Correction: In situ FRET measurement of cellular tension using conventional confocal laser microscopy in newly established reporter mice expressing actinin tension sensor

**DOI:** 10.1038/s41598-025-24977-7

**Published:** 2025-10-30

**Authors:** Junfeng Wang, Eijiro Maeda, Yuki Tsujimura, Takaya Abe, Hiroshi Kiyonari, Tetsuya Kitaguchi, Hideo Yokota, Takeo Matsumoto

**Affiliations:** 1 Biomechanics Laboratory, Department of Mechanical Systems Engineering, Graduate School of Engineering, Nagoya University, Furo-Cho, Chikusa-Ku, Nagoya, Aichi 464-8603 Japan; 2 RIKEN Center for Advanced Photonics, RIKEN, Wako, Saitama Japan; 3 Laboratory for Animal Resources and Genetic Engineering, RIKEN Center for Biosystems Dynamics Research, Kobe, Hyogo Japan; 4 Laboratory for Chemistry and Life Science, Institute of Innovative Research, Tokyo Institute of Technology, Yokohama, Kanagawa Japan

Correction to: *Scientific Reports* 10.1038/s41598-023-50142-z, published online 20 December 2023

In the original version of this Article Junfeng Wang and Eijiro Maeda were omitted as equally contributing authors. The Author Contributions section now reads:

H.Y. and T.M. conceptualized the study. T.M., H.Y., and J.W. designed the experiment. T.K. developed the FRET sensors. T.A. and H.K. developed reporter mice. J.W. and Y.T. performed the experiments. J.W., E.M., and T.M. analyzed the results. J.W. and E.M. wrote the manuscript. E.M. and T.M. finalized the manuscript. J.W. and E.M. contributed equally to this study.

The original Article has been corrected.

