# Supplementary material for: In situ FRET measurement of cellular tension using conventional confocal laser microscopy in newly established reporter mice expressing actinin tension sensor
Source: Sci Rep. 2023 Dec 20;13:22729. doi: 10.1038/s41598-023-50142-z (PMC10733408; doi:10.1038/s41598-023-50142-z)
Supplement: Supplementary file 1 — Supplementary Information. [file 41598_2023_50142_MOESM1_ESM.docx]

**Supplementary Information**

**In situ FRET measurement of cellular tension using conventional confocal laser microscopy in newly established reporter mice expressing actinin tension sensor**

Junfeng Wang^1#^, Eijiro Maeda^1#^, Yuki Tsujimura^2^, Takaya Abe^3^, Hiroshi Kiyonari^3^, Tetsuya Kitaguchi^4^, Hideo Yokota^2^, Takeo Matsumoto^1^*

1. Biomechanics Laboratory, Department of Mechanical Systems Engineering, Graduate School of Engineering, Nagoya University, Nagoya, Aichi, Japan

2. RIKEN Center for Advanced Photonics, RIKEN, Wako, Saitama, Japan

3. Laboratory for Animal Resources and Genetic Engineering, RIKEN Center for Biosystems Dynamics Research, Kobe, Hyogo, Japan

4. Laboratory for Chemistry and Life Science, Institute of Innovative Research, Tokyo Institute of Technology, Yokohama, Kanagawa, Japan.

^#^Equal contribution

*Corresponding author

Takeo Matsumoto, Ph.D., Professor

Biomechanics Laboratory, Department of Mechanical Systems Engineering, Graduate School of Engineering

Nagoya University

Furo-cho, Chikusa-ku, Nagoya, Aichi, 464-8603, Japan

E-mail: takeo@nagoya-u.jp, Tel&Fax +81 52 789 2721

This word file includes:

1. Supplementary Figure S1
2. Supplementary Figure S2
3. Supplementary Material S1

Supplementary Figure S1


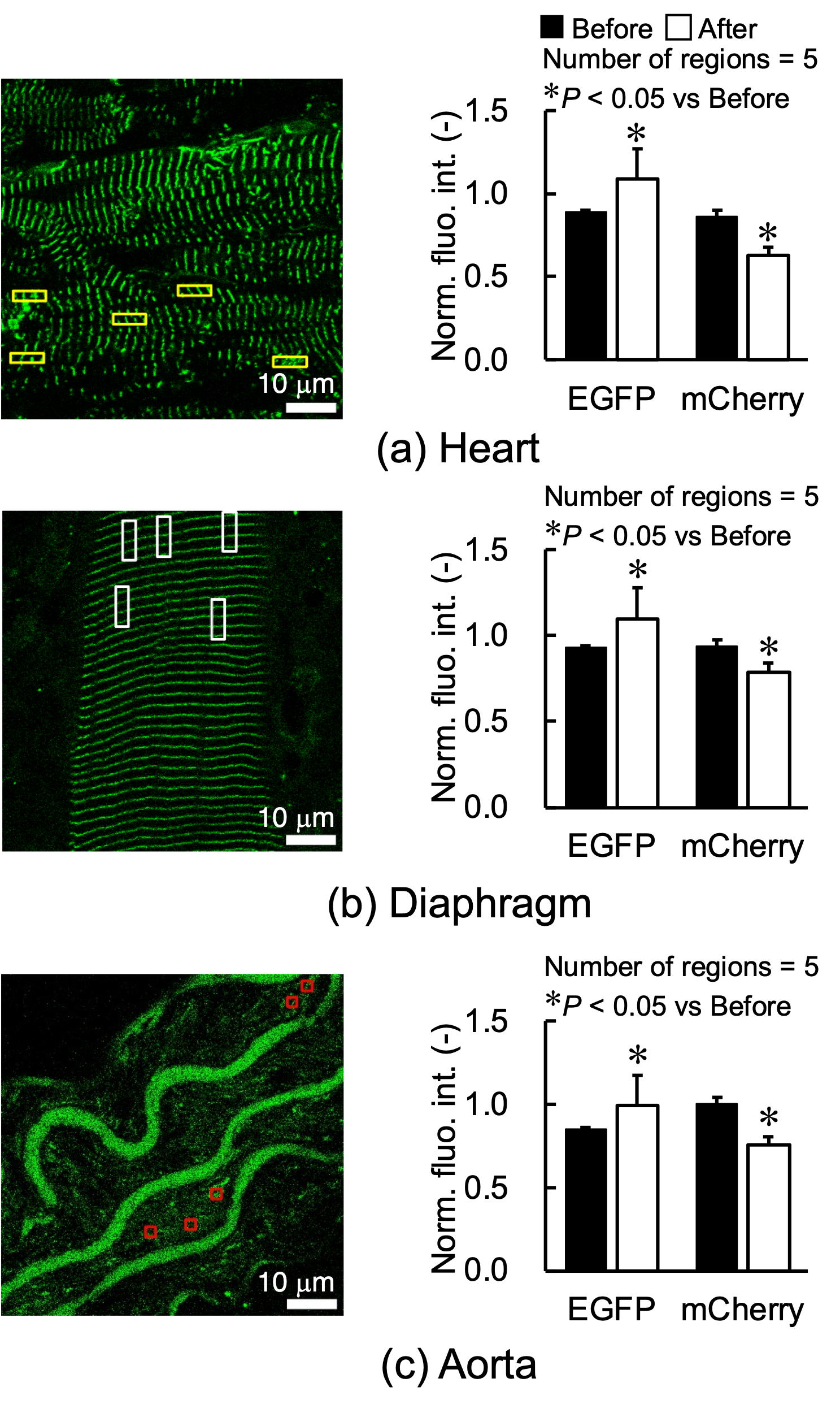


Photobleaching experiments with heart (a), diaphragm (b) and aorta (c) as shown in Figure 4. Left, fluorescent image of EGFP and ROIs (open rectangles in yellow, white or red). Right, Comparisons of normalized fluorescence intensity of EGFP and mCherry before and after photobleaching. Data were obtained from S2 tension sensors.

Supplementary Figure S2


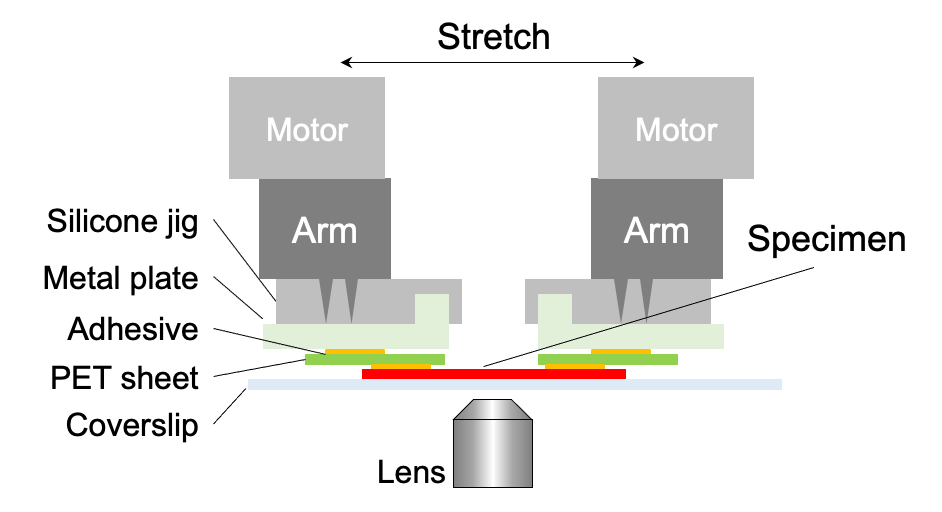


Tensile stretching apparatus and custom-made jigs for application of tensile strain to tissue explants and isolated cells^32^.

Supplementary Material S1

Bleed-through estimation

Here we estimate the effect of fluorescence bleed-through on FRET ratio measurements. Let us assume that fluorescence bleed-through from the donor fluorophore (EGFP) affects fluorescence detection of the acceptor fluorophore (mCherry) by summation of the true fluorescence intensity of the acceptor and the bleed-through from the donor, and vice versa, as follows:

$$\begin{aligned} I_{d}^{*}=I_{d}+\gamma I_{a}\#\#\#\#\#\#\#\#\#\#\left( S1 \right) \end{aligned}$$

$$\begin{aligned} I_{a}^{*}=I_{a}+\delta I_{d}\#\left( S2 \right) \end{aligned}$$

where *I_d_** and *I_d_* are the apparent and true fluorescence intensity of the donor, respectively, and *I_a_** and *I_a_* are the apparent and true fluorescence intensity of the acceptor, respectively, and γ and δ are factors between 0 and 1 corresponding the amount of fluorescence bleed-through from the acceptor to the donor and from the donor to the acceptor, respectively.

By assuming that $\gamma I_{a}$ is much smaller than $I_{d}$, the apparent FRET ratio *r*^*^ is expressed as

$$\begin{aligned} r^{*}=\frac{I_{a}^{*}}{I_{d}^{*}}\cong\frac{I_{a}+\delta I_{d}}{I_{d}}\#\left( S3 \right) \end{aligned}$$

If we look at data from the acceptor photobleaching experiment (Figure 4c), the normalized FRET ratios before and after photobleaching were 0.58 and 0.18; thus,

$$\begin{aligned} r_{\text{before}}^{*}=\frac{I_{a}+\delta I_{d}}{I_{d}}=\frac{I_{a}}{I_{d}}+\delta=0.58\#\left( S4 \right) \end{aligned}$$

$$\begin{aligned} r_{\text{after}}^{*}=\frac{I_{a}}{I_{d}}+\delta=0.18\#\left( S5 \right) \end{aligned}$$

$$\begin{aligned} \delta=0.18\left( ∵I_{a}=0 in\text{ }r_{after}^{*} \right)\#\left( S6 \right) \end{aligned}$$

The true FRET ratio *r*_true_ before photobleaching is estimated as

$$\begin{aligned} r_{\text{true}}=\frac{I_{a}}{I_{d}}=0.4\#\left( S7 \right) \end{aligned}$$

If the FRET ratio is changed from *r** to *k*r** (*k** < 1) by application of tensile stretching, the ratio in the stretched state is given as

$$\begin{aligned} k^{*}r^{*}=\frac{kI_{a}+m\delta I_{d}}{mI_{d}}\#\left( S8 \right) \end{aligned}$$

where *k* and *m* is a factor of the change in fluorescence of the acceptor and the donor by application of mechanical stretch to the FRET sensor, respectively. Because true fluorescence intensity of the acceptor decreases and that of the donor increases during stretching, *k* and *m* decrease and increase, respectively, from the initial value, 1. This reciprocal relationship is assumed to be *k* + *m* = 2. Thus, with eq. S3 we get

$$\begin{aligned} k^{*}r^{*}=\frac{kI_{a}+\left( 2-k \right)\delta I_{d}}{\left( 2-k \right)I_{d}}=k^{*}\frac{I_{a}+\delta I_{d}}{I_{d}} \end{aligned}$$

$$\begin{aligned} \frac{kI_{a}+\left( 2-k \right)\delta I_{d}}{\left( 2-k \right)}=k^{*}\left( I_{a}+\delta I_{d} \right)\#\left( S9 \right) \end{aligned}$$

Using the result from the photobleaching experiment, δ = 0.18 (from eq. S6) and *I*_a_ = 0.4*I*_d_ (from eq. S7),

$$\frac{k{0.4I}_{d}+\left( 2-k \right)0.18I_{d}}{(2-k)}=k^{*}0.58I_{d}$$

$$\frac{0.22k+0.36}{(2-k)}=k^{*}0.58$$

$$\begin{aligned} 0.22k+0.36={1.16k}^{*}-0.58k^{*}k\#\left( S10 \right) \end{aligned}$$

and finally we obtain

$$\begin{aligned} k=\frac{1.16k^{*}-0.36}{0.58k^{*}+0.22}=2-\frac{0.8}{0.58k^{*}+0.22}\#\left( S11 \right) \end{aligned}$$

Based on this relationship, FRET ratio changes with and without considering fluorescence bleed-through are estimated as in Table S1. Because the lowest normalized FRET ratio was approximately 80% in our stretching experiments with isolated tissues and cells (Fig. 6), it is sufficient to examine whether the fluorescence bleed-through can be neglected when the normalized FRET ratio decreases to 80% from an unstretched state. When the normalized FRET ratio (including fluorescence bleed-through), corresponding to a change in *r** is 0.80 (Table S1), the estimated normalized FRET ratio (excluding fluorescence bleed-through), shown as *r*_true_ change in Table S1, was 0.71, resulting less than 10% underestimation of FRET change. Accordingly, the effect of fluorescence bleed-through on the FRET ratio was not significant in this experimental setting.

| Table S1. Estimated FRET ratio changes with and without considering fluorescence bleed-through. | | | | | |
| --- | --- | --- | --- | --- | --- |
| *k** | *k* | *k*r** | *r** change | *r*_true_ | *r*_true_ change |
| (1) | (1) | (0.580) | (-) | (0.4) | (-) |
| 0.95 | 0.962 | 0.551 | 0.95 | 0.371 | 0.927 |
| 0.90 | 0.921 | 0.522 | 0.90 | 0.342 | 0.855 |
| 0.85 | 0.877 | 0.493 | 0.85 | 0.313 | 0.782 |
| 0.80 | 0.830 | 0.464 | 0.80 | 0.284 | 0.71 |

Values in parentheses in the top row represent the unstretched state, where *k***r** and *r*_true_ are taken from the acceptor photobleaching experiment (Figure 4). When *k** is arbitrarily given, *k* and *m* are calculated using eq. S11 and *k* + *m* = 2, *k***r** is calculated with *k*, *m*, and δ using eqs. S6-8. An *r** change (equal to *k** as expected) is calculated with *k***r** at the given *k** divided by *k***r** in an unstretched state (= 0.58). *r*_true_ is given with *k*, *m*, and eqs. S7-8 where δ = 0, estimating no bleed-through, and *r*_true_ is obtained from *r*_true_ at the given *k** divided by *r*_true_ in an unstretched state (= 0.4).
